# Heating, ventilation and air conditioning (HVAC) in intensive care unit

**DOI:** 10.1186/s13054-020-02907-5

**Published:** 2020-05-06

**Authors:** Sai Saran, Mohan Gurjar, Arvind Baronia, Vijayalakshmi Sivapurapu, Pralay S. Ghosh, Gautham M. Raju, Indubala Maurya

**Affiliations:** 1 Department of Critical Care Medicine, Super Speciality Cancer Institute and Hospital, Lucknow, Uttar Pradesh 226002 India; 2 Department of Critical Care Medicine, Sanjay Gandhi Postgraduate Institute of Medical Sciences (SGPGIMS), Lucknow, Uttar Pradesh 226014 India; 3 Department of Anaesthesiology, Indira Gandhi Medical College and Research Institute, Puducherry, 605 006 India; 4 Department of Critical Care Medicine, Tata Medical Centre, Kolkata, West Bengal 700156 India; 5 Department of Critical Care Medicine, Manipal Hospitals, Benguluru, Karnataka 560017 India; 6 Department of Anesthesiology, Super Speciality Cancer Institute and Hospital, Lucknow, Uttar Pradesh 226002 India

**Keywords:** Heating, ventilation and air-conditioning, Intensive care unit, Indoor air quality, Ventilation, Infection control

## Abstract

The aim of this review is to describe variation in standards and guidelines on ‘heating, ventilation and air-conditioning (HVAC)’ system maintenance in the intensive care units, across the world, which is required to maintain good ‘indoor air quality’ as an important non-pharmacological strategy in preventing hospital-acquired infections. An online search and review of standards and guidelines published by various societies including American Institute of Architects (AIA), American Society of Heating, Refrigerating and Air-Conditioning Engineers (ASHRAE), Centers for Disease Control and Prevention (CDC), Department of Health Estates and Facilities Division, Health Technical Memorandum 2025 (HTM) and Healthcare Infection Control Practices Advisory Committee (HICPAC) along with various national expert committee consensus statements, regional and hospital-based protocols available in a public domain were retrieved. Selected publications and textbooks describing HVAC structural aspects were also reviewed, and we described the basic structural details of HVAC system as well as variations in the practised standards of HVAC system in the ICU, worldwide. In summary, there is a need of universal standards for HVAC system with a specific mention on the type of ICU, which should be incorporated into existing infection control practice guidelines.

## Introduction

Heating, ventilation and air-conditioning (HVAC) has a pivotal role in determining infection rates in the intensive care unit (ICU), apart from its primary purpose of providing comfortable living and safe environment for the patients, ICU staff and visitors [1, 2]. Essential functions of HVAC system includes heating (adding heat to raise or maintain temperature), cooling (removing heat to lower or maintain temperature), humidifying (adding water vapour), dehumidifying (removing water vapour) in order to maintain the moisture content of the air, filtering (removing dust particles, biological contaminants like bacteria, viruses and fungi), ventilating (air change rates between outdoor) and air distribution (velocity, flow pattern, direction of movement and distribution patterns) [1, 3]. These functions result in air conditioning, which aid in the prevention of contamination and cross-contamination and environmental protection along with operator protection [1, 2]. Maintaining good indoor air quality (IAQ) is an important non-pharmacological strategy in preventing hospital-acquired infections [4].

The objective of this article is to review various worldwide available standards, guidelines and recommendations on HVAC system which mentions required standards in the ICU (Table 1**)** [5–15]. In order to understand the terminology used in describing the HVAC system, we also described the basic types and constituents of HVAC system along with the maintenance and monitoring of its function [3].

**Table 1:** Worldwide professional societies and government bodies recommending HVAC system focusing on ICU

Country	Professional aociety/government body	Reference
Australia	• Queensland Health Facility Guideline (QHFG)	[5]
Germany	• Verein Deutscher Ingenieure (VDI)	[6]
India	• Indian Society of Critical Care Medicine (ISCCM)	[7, 8]
United Arab Emirates	• Dubai Health Authority (DHA)	[9]
UK	• Department of Health Estates and Facilities Division (DHF),	[10–12]
	• Health technical memorandum (HTM 2025)	
USA	• American Institute of Architects (AIA)	[13–15]
	• American Society of Heating, Refrigerating and Air-Conditioning Engineers (ASHRAE)	
	• Centers for Disease Control and Prevention (CDC)	

## Clinical importance of HVAC system

Airborne diseases have been linked to poorly functioning HVAC system: improper function of temperature control, humidity control, air distribution and filtration of HVAC systems [16–28].

Lutz et al. reported an outbreak of *Aspergillus* infection among inpatients who had been operated in the same operating room in a 12-day period. Their investigations revealed ≥ 3 μm bio-aerosols in the operating room through air sampling, which was considered as a surrogate marker of *Aspergillus* conidia [17]. They also found contaminated diffusers, duct work and other duct materials which were cultured for *Aspergillus* species. Similar outbreaks of *Aspergillus* infections were reported in health care systems linked to air conditioning plants [18].

Air samples positive for methicillin-resistant *Staphylococcus aureus* (MRSA) were reported by Rutala et al. in a burn unit indicating the transport of such infections through air [19]. Aerial dissemination of antibiotic-resistant *Acinetobacter anitracus* in an ICU was identified through settle plates by Allen et al. [20]. Later on, such aerial dissemination of carbapenem-resistant *Acinetobacter* species was proven by Das et al. from the movement of heavily contaminated bed curtains in the hospital [21]. Similarly, *Clostridium difficile* spores were recovered from air vents in a 22-month surveillance study suggesting aerial spread of infections [22]. Multiple outbreaks were reported for infections caused by *Legionella* linked to HVAC systems including the cooling towers, ductwork and filters [23, 24].

Lee suggested that diseases like tuberculosis require ‘three-level’ hierarchy to control the spread of disease in hospitalized patients: first, medical-administrative role in early diagnosis, isolation and treatment; second, environmental aspect by reducing the concentration of airborne bacilli by increasing number of air changes per hour (ACR) and single-pass ventilation systems where 100% supplied air is exhausted to avoid re-circulation; and the third level, being the use of personal protective equipment [25].

There are reports of severe acute respiratory distress syndrome (SARS) outbreaks in hospital wards from Hong Kong suggesting aerial dissemination in viral infections like coronavirus (SARS-CoV) which can lead to epidemics, indicating that there exists a correlation between building factors related to air circulation and rate of occurrence of infections [26, 27]. The functional aspects of HVAC system like relative humidity and temperature were found to have a significant correlation with nosocomial infection rates especially in ICUs and geriatric population [28].

There are various factors which play a role in the transmission of airborne diseases such as particle characteristics: size (droplet ≥ 5 μm; airborne pathogen ≤ 5 μm), type, life span, distance travelled and the surrounding environment along with architectural design, apart from host risk factors [16, 29]. Once bacteria, moulds or allergens enter a building, air handling systems (HVAC) control their spread, aid in their removal from the system in both droplet-borne (pertussis, influenza, coronavirus, *Staphylococcus aureus*) and airborne diseases (tuberculosis, *Aspergillus*, norovirus, varicella zoster virus) [16, 29–31]. This is achieved either by ‘diluting’ the pathogen (dilution ventilation) or by removing the pathogen (exhaust ventilation) [3, 16, 30, 32, 33]. An improperly maintained HVAC system can be a continuing source of contamination, an example of which is the growth of moulds and other fungi in damp and wet surfaces such as cooling coils, humidifiers, condensate pans and filters [17–23]. Poorly designed and maintained HVAC systems in the ICU can lead to ‘sick building syndrome’ (SBS), characterized by suffocation which can lead to decreased staff performance in addition to various hospital-acquired infections and occupational hazards [34].

## Components of HVAC system

There are three basic components: (1) outdoor air intake and air exhaust ducts and controls, (2) air handling units (AHU), and (3) air distribution systems.

### Outdoor air intake and air exhaust ducts and controls

Dampers are used to cut off central air-conditioning to unused rooms and for regulating the air supply room-by-room. These can play a role as economizers that can be placed at supply, relief and return components of HVAC system. Louvres are used for protection from water infiltration [3].

### Air handling units (AHU)

**Fig. 1:**
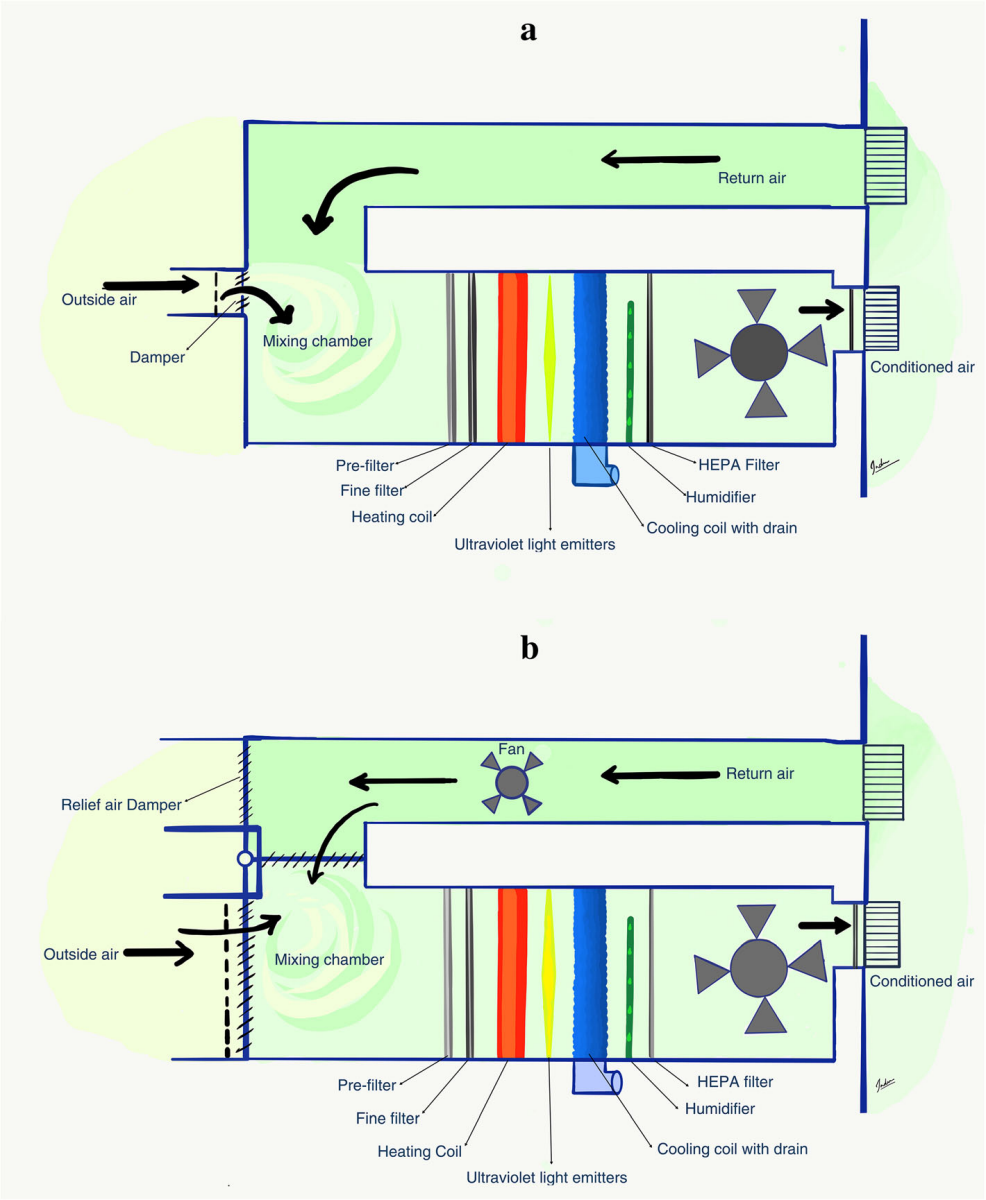
**a** Basic structure of Air-conditioning plant. **b** Air-conditioning plant with economizer cycle

Its function is to take in outside air, re-condition it and supply as fresh air [3]. The integral components of an AHU are shown in Fig. 1a and b.

#### Heat exchangers and chillers (humidity modification system)

Heat exchangers transfer heat (energy) from one fluid to another, which is being physically separated. Chillers are located in the basement (cooled by water) or on the roof (cooled by air). Chillers remove heat from the water, and then this water is used as a refrigerant to remove heat from the building and dehumidify air.

#### Compressor

This is located at the outdoor condenser unit which supplies air or other gases at increased pressure to the AHU.

#### Fans

Supply fans provide energy to drive the air through the system. Centrifugal fans generate adequate pressure without noise and are more commonly used than axial fans. Return fans handle air before the exhaust fans, operating in conjunction with supply fans to balance the amount of air supplied to and exhausted (Fig. 1b). All heat recovery devices create a resistance (pressure drop), against which the fan has to operate. Exhaust fans discharge air to the exhaust duct or directly outdoors.

#### Filters

These are placed to purify the incoming air into the system. Three main characteristics of filters are the efficiency in removing various sized particles in air, create resistance to flow and their dust holding capacity [3]. Filters are rated based on their Minimum Efficiency Reporting Value (MERV) rating. This ranges from 1 to 20 with higher values suggesting better filter efficiency. MERV 10 will be able to filter 1–3 μm size, whereas MERV > 13 will be able to filter particles of 0.3 to 1 μm. Medical facilities usually require MERV 14 to 16. Those filters with MERV ≥ 17 are called high-efficiency particulate air (HEPA) filters [3]. HEPA filters with MERV 17 rating have an efficiency of 99.97% against 0.3 μm size particles. For clean rooms like body implants manufacturing site, MERV should be 20 (filtration efficiency of 99.999%). Usually, in an AHU, there will be a pre-filter with low to medium efficiency acting as ‘roughing filter’ removing large particulate matter and many microbes (MERV ≤ 4). They are called ‘general filters’ graded from G1 to G4 [10]. They have efficiency up to 70% down to 10 μm sized particle. Their role is very good in arrestance, ability to arrest larger particles like dirt, lint, hair and dust. Following filtration by pre-filter, air subsequently has to pass through an additional filter bank (second bank filter) with 99% efficiency for particles up to 5 μm, with pressure drop not exceeding 20 mm water gauge (WG). These are called as ‘fine filters’ graded from F5 to F9 [10]. Following this, air is passed through HEPA with 99.97% efficiency removing particles of 0.3 μm diameters and delivered to each zone of the building (Fig. 1a, b). The pressure drop at this stage should not exceed 50 mm WG. For particles sized 0.3 μm, pre-filters have 30–35% efficiency, whereas post-filters (final filters: HEPA) have 99.97% efficiency. Inline disposable pre-filters are used to increase the life of the HEPA filter. This concept of progressive filtration increases the life of HEPA filters [3]. During outbreaks, the concentration of airborne *Aspergillus* conidia in patient care areas can be up to 100 conidia/m^3^ (CFU/m^3^), whereas in rooms with HEPA, filtration the counts below 0.1 CFU/m^3^ were reported [35]. Guidelines for infection control in health care facilities show that the filtration system with HEPA filters is adequate for most patient care areas [36]. These filters are at least efficient for removing particles sized more than 0.3 μm in diameter [3].

#### Drains and drain pans

It allows water to be drained and discharged.

#### Ultraviolet (UV) light emitters or ultraviolet germicidal irradiation (UVGI)

Ultraviolet light is used as a germicidal irradiation (UVGI) agent to reduce the virulence of the microorganisms. This is usually used in the exhaust air ducts of HVAC system to supplement HEPA filtration (Fig. 1a, b).

#### Noise attenuators

These help in the reduction of noise from various motors (fans, coolers, heaters, humidifiers) and other equipment in HVAC system.

### Air distribution systems

It can be of full fresh air or single-pass system (displacement ventilation) or the traditional re-circulation type (mixing ventilation) systems **(**Fig. 1a, b), where recirculated air requires adequate filtration [3, 37, 38]. Displacement ventilation has advantages like better acoustics and less noise, low pressure drops, and low energy consumption, requires a lower grade of filtration and has high ventilator efficiency as 100% fresh air is delivered to the occupants [38]. The effectiveness of 100% fresh air system combined with UVGI is almost similar to the use of re-circulated air coupled with the use of HEPA filtration, provided an adequate number of ACH were maintained [31]. The energy cost of the latter is lesser when compared to the former [31]. Air velocity and distribution also play an important role in air distribution system. The preferential path is that of laminar airflow pattern from clean to dirty spaces diverting the particles to the exhaust grillers rather than turbulent pattern [39].

#### Supply and return air ducts

This is usually made up of non-corrosive material. Return air duct carries air from conditioned space to the mixing chamber.

## Air distribution in special zones—protective isolation rooms

ICUs require isolation of patients to protect them from the external environment (positive pressure) and also need to prevent the spread of infections from the patient to the external environment (negative pressure). Two room pressurization control methods are made by creating airflow differentials by altering the supply, return and exhaust air proportions (Table 2). Both methods of control require visual or other types of indicating system to monitor them for their efficiency as they have the tendency to lose their calibration over time [3].

**Table 2:** Differences between positive and negative pressure rooms

Characteristic	Positive pressure system	Negative pressure system or airborne infection isolation room (AIIR)
Purpose	To create a protective environment to the patient to avoid acquiring any airborne infection (does not require an ante-room).	To create a protective environment to the healthcare providers as well as other patients in the ICU (requires an ante-room).
Type of patients requiring isolation	Burns, post-transplant, febrile neutropenia (also for patients in operating rooms)	Tuberculosis, swine flu, COVID-19 and other airborne viral diseases
Direction of airflow	Outside the room (away from the patients)	Inside the room (towards the patient)
Pressure	More than 2.5 Pa preferably + 8 Pa (ideal)	Less than 2.5 Pa
Supply air	More than the sum of return and exhaust air	Less than the sum of return and exhaust air
Recirculation	90–95%	80–90% (if required)
Air change per hour	> 20	≥ 12
Filtration efficiency	Supply: 99.97% at 0.3 μm DOP All supply air must pass through HEPA filters	Supply: 90% (dust spot test) All supply air to be exhausted without recirculation HEPA (99.97% at 0.3 μm DOP) must be used on the supply side if recirculation is used. HEPA is required on the exhaust side too, when exhausting air to the outside is not practical

*DOP* dioctylphthalate particles of 0.3 μm diameter, *Pa* Pascal, *HEPA* high-efficiency particulate air filter, *COVID-19* coronavirus disease 2019

## Monitoring and maintenance of the HVAC plant

Maintenance programmes can be of two types: reactive maintenance, implemented when there is any malfunction of the plant, or planned preventive maintenance (PPM) programme, done at periodic intervals without interrupting patient care. The zone of ICU comes under ‘very high-risk category (category A)’ in which the standards of maintenance are critical [5]. To ensure this, apart from imparting knowledge of the plant to the ICU working team, it is critical that the engineer makes these components easily accessible, especially components like access doors for drain pans. There should be clear labelling of each component like AHU, outdoor air (OA), supply air (SA), return air (RA) and exhaust air (EA), each with arrows noting the proper flow directions (Table 3).

**Table 3:** Maintenance suggestions for HVAC system

S. N.	HVAC system maintenance
1	Ensure proper labelling of parts of HVAC plant including the direction of airflow mentioning OA, SA, RA and EA.
2	Outside air intakes must be examined for any dust and moisture.
3	Drain pans and pipes checked for any accumulation of condensate water.
4	Clean condenser and evaporator coils.
5	Indoor air quality should be frequently checked usually every 6 months.
6	HEPA filter efficiency tests and efficiency rating label every 6 months.
7	Lubricate motors bearings, fans and moving parts.
8	Continuous monitoring of the humidity and temperature in ICU.

*OA* outdoor air, *SA* supply air, *RA* return air, *EA* exhaust air

### Air quality standards

Indoor air quality should be frequently checked usually every 6 months [14, 15]. Air quality can be checked by active methods or passive methods. Index of microbial air contamination (IMA) which is performed using 1/1/1 scheme (1 h of exposure at 1 m from the floor and at least 1 m away from the wall) [40]. Estimation of microbial load is done by the formula: *B* = 1000/*NRT*, where *B* is the bacteria-containing particles/mm^3^, *N* number of colonies on the plate, *R* rate of sampling and *T* time given for sampling [40]. There are certain acceptable IMA limits, which vary according to the area of the hospital tested, such as the maximum acceptable IMA is 5 for ultra-clean rooms, isolation rooms and operating room for joint replacements, whereas in ICU and dialysis rooms, IMA is acceptable up to 25 [40]. Apart from testing for microbial loads, air is also tested for moisture content and its relation to air temperature and energy using a psychrometric chart [3].

### Monitoring HEPA filter performance

HEPA filter efficiency is assessed by various tests such as the dispersed oil particulate (DOP test) test, dust spot test and weight arrestance test [41].. Once the filter is tested, its efficiency percentage and pressure drop across the filter need to be mentioned on the filter as ‘efficiency rating label’ [41]. For optimal performance, HEPA filters also need to be tested every 6 months, apart from indoor air quality monitoring [14, 15].

### Maintenance of the HVAC plant

The interaction between fans and filters for the maintenance of pressure and flow directions in various places causes unavoidable wear and tear of the plant. This process requires periodic air vent monitoring, odour monitoring, and replacement of filters as per the manufacturer’s recommendations along with ‘filter forensics’ which include recovery of microbiota from residual HVAC filters [42]. Other than filter testing, annual maintenance of fans, bearings and belts, fixing leaks in the cabinet and the supply duct along with performance monitoring of chilled water distribution systems (heat exchangers) are all necessary (Table 3) [3].

## Recommended standards for HVAC system in the ICU from various professional societies and government bodies

HVAC for a sterile area differs from that of a comfortable area in terms of created pressure differentials, air changes per hour (ACH), air velocity, air distribution patterns and filtration apart from comfort parameters like temperature and relative humidity [3]. Even in sterile areas, there are varying requirements in different areas, such as in central sterile supplies department (CSSD), ICUs, operating rooms and implant manufacturing sites [3, 32]. In ICUs too, there is a requirement of different standards based on the patient population (general, neonates, burns, etc.) (Table 4). Recommendations and standards from American Institute of Architects (AIA), American Society of Heating, Refrigerating and Air-Conditioning Engineers (ASHRAE), Centers for Disease Control and Prevention (CDC), Department of Health Estates and Facilities Division, Health Technical Memorandum (HTM 2025) and Healthcare Infection Control Practices Advisory Committee (HICPAC) are being followed in the construction and maintenance of the majority of healthcare systems [5–15]. Only a few of these mention ICU standards for HVAC system, which are presented and compared in Table 4. There are a lot of variations in the recommended temperature, airflow patterns, relative humidity, pressurization relative to the surroundings, filtration standards and air changes per hour (ACH) in these standards.

**Table 4:** Comparison of various standards for HVAC in ICUs across the world

Country	Recommendation society/association (reference)	Temperature	Relative humidity	Filtration	Pressurization	Air change (outside air/total) per hour [ACH]	Specific highlights/key differences
**Type of ICU—general**							
Australia	QHFG [5]	21–24 °C	30–60%	G4–F8	Positive	2/6	Filtration: Standards varied from MERV 7–8 to MERV 15. Few recommend HEPA filters (MERV ≥ 17). Pressurization: Positive pressure inside the ICU zone is recommended by Australian, UAE and UK societies, while neutral pressure is recommended in Germany, India, USA and recent UK HTM 2025. Temperature: Wide range varying from 16 to 25 °C. Relative humidity: Majority suggests 30–60% range whereas Indian and German recommendations remain silent. ACH: HTM 2025 (UK) strongly discourages the use of re-circulation type HVAC, presumably to avoid recirculation of airborne pathogens. Air distribution pattern: There exists no specific recommendation of air distribution pattern
Germany	VDI [6]	–	–	F9	Neutral	–	
India	ISCCM [7, 8]	16–25 °C	–	99% efficiency till 5 μm	Neutral	2/6	
UAE	DHA [9]	21–24 °C	30–60%	HEPA	Positive	2/6	
UK	DHF [10, 11]	18–25 °C	–	F7	Positive^a^	10 (total)^b^	
UK	HTM 2025 [12]	20–22 °C	40–60%	–	Neutral	100% FA	
USA	AIA [13]	21–24 °C	30–60%	–	Neutral	2/6	
USA	ASHRAE [14]	21–24 °C	30–60%	–	Neutral	2/6	
**Type of ICU—burn**							
Australia	QHFG [5]	21–32 °C	30–95%	G4–F8	Positive	3/6	Filtration: Australian recommendations suggest filtration similar to general ICUs whereas the USA recommends HEPA filtration of incoming air. Pressurization: Positive pressure isolation is recommended by Australia and the USA, whereas the rest have no mention. Temperature: Higher range (21–32 °C), in comparison to general ICU, is recommended by QHFS. Relative humidity: Higher range (up to 95%), in comparison to general ICU, is recommended by ASHRAE. Air distribution pattern: It should be ‘laminar’, as recommended by ASHRAE.
USA	ASHRAE [14]	–	40–60%	HEPA	Positive	3/6	
**Type of ICU—neonate**							
Australia	QHFG [5]	22–26 °C	30–60%	G4–F8	Positive	2/6	Filtration: There is no mention of air filtration standards by the USA whereas Australia suggests similar filtration standards as general ICUs. Pressurization: Australian recommendations suggest positive pressure whereas the USA recommends neutral pressure Temperature: Slight higher range (22–26 °C) is recommended in comparison to other ICUs. Relative humidity: Neonates having similar RH as in adults is a concern Air distribution pattern: There exists no specific recommendation of air distribution pattern
USA	ASHRAE [14]	22–26 °C	30–60%	–	Neutral	2/6	

*ASHRAE* American Society of Heating, Refrigerating and Air-Conditioning Engineers, *AIA* American Institute of Architects, *VDI* Verein Deutscher Ingenieure (German engineers association), *UAE* United Arab Emirates, *DHA* Dubai Health Authority, *DHF* Department of Health Estates and Facilities Division, *HTM* Health Technical Memorandum, *QHFG* Queensland Health Facility Guideline, *ISCCM* Indian Society of Critical Care Medicine, *HEPA* high-efficiency particulate air filter, *G4* (general filter) filters for coarse dust particles which are efficient for particles ≥ 10 μm (equal to MERV 7 and 8 ASHRAE), *F8 and F9* (fine filters) filters for fine particles which efficient for particles ≥ 1 μm (F8 equal to MERV 14, F9 equal to MERV 15–16), *F7* fine filter with up to 99% efficiency till 5 μm, *MERV* Minimum Efficiency Reporting Value, *FA* fresh air

^a^Isolation room may be of negative pressure

^b^Where highly infective or vulnerable patients like burns and immune deficiency are regularly admitted and at least 15 ACH are recommended

### Temperature

There is a wide variation in the recommended temperature from 16 °C to 25 °C in general ICUs [5–15]. Australian, UAE and US recommendations mention the temperature of 21–24 °C; whereas the UK recommends 18–25 °C and India recommends 16–25 °C, German recommendations are silent on this aspect. Australian recommendations suggest a higher range of temperature (21–32 °C) in burns unit, whereas others remain silent on this aspect. Australia and the USA suggest temperature in between 22 and 26 °C in neonatal ICUs and the rest of the societies does not mention any specific range for neonatal ICU.

### Relative humidity

Australian, UAE and US recommendations suggest relative humidity (RH) of 30% to 60% whereas the UK recommends 40–60% [5–15]. German and Indian recommendations are silent on this crucial aspect. Higher humidity range (30–95%) was suggested in burns ICU probably to enhance wound healing by Australian recommendations, whereas the USA recommends 40–60%. There is no mention about RH which plays a definitive role in infection control and wound healing in burns ICUs by others. Australian and US guidelines recommend 30–60% RH in neonatal ICUs whereas in others, there is no such specific recommendation.

### Filtration

Regarding filtration, there is wide variation, with the Australian recommendations suggesting progressive filtration with general (G4) followed by fine filter (F8), which is equivalent to Minimum Efficiency Reporting Value (MERV) 7 and 8 ASHRAE, whereas German recommendations suggest filtration through fine filter (F9), which is equivalent to MERV 15–16 [5, 6]. Indian guidelines mention filtration up to 99% efficiency till 5 μm, which is similar to the UK standards of using fine filtration with F7 [7, 8, 10–12]. The use of HEPA filtration was recommended only by Dubai Health Authority in general ICU [9]. With regards to burns ICU, Australian recommendations suggest the same level of filtration as general ICU, whereas the USA recommends filtration through HEPA filters [5].

### Pressurization

Australian, UK and Dubai Health Authority (DHA) recommendations suggest positive pressurization in general ICUs, whereas others (including UK HTM 2025) recommend neutral pressure in this area [5, 9–12]. Australian and US recommendations suggest positive pressurization in burns ICUs, whereas positive pressurization is recommended in neonatal ICU by Australia only [5, 13–15].

### Air change (outside air/total) per hour

Another aspect to consider is the air changes per hour (ACH), which is the volume of air entering the room per hour—the higher the ACH, the shorter is the time required for efficient particulate removal [3, 39]. Most of the recommendations suggest a total of 6 ACH, out of which two exchanges should be with outside air, whereas operating rooms require a minimum of 20 ACH [5–15], whereas recommendations from the UK suggest ACH should be ten, which could be increased to 15 if the patients are at high risk of spreading infections or immunocompromised [10, 11]. UK Health Technical Memorandum (HTM 2025) suggests the use of 100% fresh air system and discourages the usage of re-circulation-based systems [12].

### Air distribution pattern

As the number of air changes per hour (ACH) increases, the chances of cross-infection decrease, provided there exists an optimized design between the contaminant source and the exhaust being described as ‘confirming to the path’ principle [43]. When the path is interrupted by air streams, the pathogen is likely to migrate to other places of the room. This creates the importance of air distribution pattern which is suggested only by ASHRAE in burns ICU. The placement of inlets (diffusers) which deliver air also forms an important aspect of air distribution pattern, and they should not be placed over the heads of occupants (patients or staff). Careful thought must be given to the size and location of the air inlets so that turbulence is avoided in its path [44].

Despite well-known effects of HVAC in infection control, there are significant differences which are highlighted among these standards. It is concerning that even in the guidelines on prevention of hospital-acquired pneumonia, not much importance has been given to developing HVAC design and maintenance standards as an infection prevention strategy [45, 46].

Although no real consensus exists worldwide, the use of 100% fresh air system, directed room airflow (from clean to dirty) preferably laminar pattern of air distribution pattern with high frequency of ACH (≥ 12/h) along with pressurization based on the patient cohort along with the use of HEPA filtration of the incoming air could be considered as a universally accepted measure to reduce infections in hospitals [5–15, 27, 30, 31, 36, 43].

## Coronavirus disease 2019 (COVID-19) and HVAC system

With the recent pandemic of coronavirus (COVID-19), the importance of HVAC system in infection control is further highlighted by various interim guidelines (updated till 20 March 2020) [47–51] **(**Table 5**)**. CDC recommends to isolate patients with suspected COVID-19 in airborne infection isolation rooms (AIIRs) with a minimum ACR of 6 per hour (12 ACR for new construction or renovation), along with the use of HEPA filtration of the incoming air, if re-circulated [47]. World Health Organization (WHO) recommends COVID-19 patients to be isolated in an adequately ventilated negative pressure rooms with a minimum of 12 ACH, especially if aerosol-generating procedures are planned [48, 49]. Similar recommendations are proposed by Surviving Sepsis Campaign guidelines released by the European Society of Intensive Care Medicine (ESICM) and Society of Critical Care Medicine (SCCM) [50]. European Centre for Disease Prevention and Control remains silent on the above aspects but gives importance to frequent cleaning and maintenance of HVAC systems [51].

**Table 5:** Leading organizations/societies recommendations for HVAC system in the management of COVID-19 patients [updated till 20 March 2020]

	Name of the organization/society (reference)			
	CDC [47]	WHO [48, 49]	ESICM/SCCM [50]	ECDC [51]
Pressurization	Negative	Negative	Negative	No mention
Temperature	No mention	No mention	No mention	No mention
Relative humidity	No mention	No mention	No mention	No mention
Air change (outside air/total) per hour (ACH)	Minimum of 6, while 12 in new construction or renovation	At least 12	At least 12	No mention
Filtration	HEPA filtration if re-circulated	No mention	HEPA filtration if re-circulated	No mention
Air distribution pattern	Appropriate directionality	Controlled direction of airflow	No mention	No mention
Special comments	–	–	–	Increase frequency of cleaning and maintenance of HVAC systems should be considered

*WHO* World Health Organization, *CDC* Center for Disease Control and Prevention, *ESICM* European Society of Intensive Care Medicine, *SCCM* Society of Critical Care Medicine, *ECDC* European Centre for Disease Prevention and Control, *HEPA* high-efficiency particulate air filter, *COVID-19* coronavirus disease 2019

There is a lack of information on various other integral aspects of HVAC system, including temperature and relative humidity, in all these recent guidelines. In fact, some earlier studies reported that the viability of coronavirus causing severe acute respiratory distress syndrome (SARS CoV) on smooth surfaces which was over 5 days at 22–25 °C and relative humidity of 40–50% was rapidly lost (> 3 log_10_) at higher temperatures (38 °C) and higher relative humidity (> 95%) [52, 53]. But the feasibility and practicability for incorporating this information is not easy in the clinical practice.

## Recent advances in HVAC system

An efficient HVAC system adjusts its various components such as heating, cooling, air filtration, air distribution, air flow rate and air exchange rates in accordance with the environment, clinician and patient needs. Modern systems have the ability to control IAQ by assessing temperature, carbon dioxide (CO_2_) concentration, humidity and airflow rates and adjusting according to the work environment. An ‘intelligent HVAC’ can sense the interaction between users and space and modify the working environment according to the needs, with minimal use of thermal or electrical energy thereby contributing to energy conservation aiming at creating a ‘green hospital’ [54, 55]. In view of the varied requirement of ventilation needs for various places and occupants, further advancement in this field is a move towards ‘personalized ventilation (PV)’, where the supplying air terminal devices (ATD) are located close to the breathing zone of the occupants who can regulate the temperature and humidity and thereby their own inhaled air quality [56].

## Conclusion

Knowledge and understanding of proper functioning of HVAC systems is crucial for critical care physicians, infection control committee members and the administrators to provide optimal safety and comfort to the ICU patients, staff and visitors, while reducing the spread of airborne infections. With the continuous advancement of technology, there exists an urge for clear and consistent universal standards for HVAC system with a specific mention on the type of ICU like general, neonatal, and burns, which should be updated and incorporated into existing infection control practice guidelines.

## Data Availability

All data generated or analysed during this study are included in this published article.

## References

[1] Bartley JM, Olmsted RN, Haas J (2010). Current views of health care design and construction: practical implications for safer, cleaner environments. Am J Infect Control.

[2] Luongo JC, Fennelly KP, Keen JA (2016). Role of mechanical ventilation in the airborne transmission of infectious agents in buildings. Indoor Air.

[3] Robert McDowall. Fundamentals of HVAC systems, 1st edition. Robert McDowall, editor. Atlanta, GA 30329, USA: Elsevier Ltd; 2006.

[4] Curtis LT (2008). Prevention of hospital-acquired infections: review of non-pharmacological interventions. J Hosp Infect.

[5] Queensland Health Facility Guideline. 2010. Reference to web site: https://www.health.qld.gov.au/_data/assets/pdf_file/0029/150977/qh-gdl-374-10.pdf. Accessed on 25 Nov 2019.

[6] German guidelines for hospital ventilation and air conditioning, VDI 2167 (Verein Deutscher Ingenieure 2004 December). Reference to web site: https://m.vdi.de/uploads/tx_vdirili/pdf/9875770.pdf. Accessed 25 Nov 2019.

[7] Rungta N. Guidelines Committee ISCCM. ICU Planning and Designing in India– Guidelines 2010. Reference to web site: https://isccm.org/pdf/Section1.pdf. Accessed 25 Nov 2019.

[8] Rungta N, Zirpe KG, Dixit SB (2020). Indian society of critical care medicine experts committee consensus statement on ICU planning and designing, 2020. Indian J Crit Care Med.

[9] Health Facility Guidelines 2012 Planning, Design, Construction and Commissioning. Dubai Health Authority. Reference to web site: https://www.dha.gov.ae/Documents/Regulations/Guidelines/Health%20Facility%20Guidelines%20_Planning%20Design%20Construction%20and%20Commissioning.pdf Version 2.0 Accessed 25 Nov 2019.

[10] Department of Health / Estates and Facilities Division. Health Technical Memorandum 03–01: Specialised ventilation for healthcare premises. Part A - Design and installation 2010. KenHolmes(au).Referencetowebsite:https://assets.publishing.service.gov.uk/government/uploads/system/uploads/attachment_data/file/144029/HTM_03-01_Part_A.pdf. Accessed 12 Dec, 2019.

[11] Standards of intensive care units. Intensive Care Society 1997. Reference to website: http://www.md.ucl.ac.be/didac/hosp/architec/UK_Intensive_care.pdf. Accessed 12 Dec 2019.

[12] National Health Service. Design considerations: ventilation in healthcare premises. Health technical memorandum 2025. London: National Health Service Estates; 1994. Reference to website:https://www.mintie.com/assets/img/education/NHS%20Estates%20-%20HVAC.pdf. Accessed 13 Dec 2019.

[13] AIA’s Guidelines for Design and Construction of Health Care Facilities book which approaches to hospital design in terms of architectural, mechanical and electrical engineering points of views (American Institute of Architects 2006) Reference to web site: https://www.fgiguidelines.org/wp-content/uploads/2015/08/2001guidelines.pdf. Accessed 20 Nov 2019.

[14] The American Society of Heating Refrigerating and Air-Conditioning Engineers. ASHRAE 2006. Reference to web site: http://www.ashrae.org. Accessed 25 Nov 2019.

[15] Centers for Disease Control and Prevention. Guidelines for environmental infection control in health-care facilities: recommendations of CDC and the Healthcare Infection Control Practices Advisory Committee (HICPAC) (2003). Updated in 2017. Reference to web site: https://www.cdc.gov/infectioncontrol/guidelines/environmental. Accessed 20 Nov 2019. 12836624

[16] Fernstrom A, Goldblatt M. Aerobiology and its role in the transmission of infectious diseases. J Pathog. 2013;493960 10.1155/2013/493960. 10.1155/2013/493960 PMC3556854 23365758

[17] Lutz BD, Jin J, Rinaldi MG (2003). Outbreak of invasive Aspergillus infection in surgical patients, associated with a contaminated air-handling system. Clin Infect Dis.

[18] Cristina ML, Sartini M, Spagnolo AM (2009). Health care-acquired aspergillosis and air conditioning systems. J Prev Med Hyg.

[19] Rutala WA, Katz EB, Sherertz RJ, et al. Environmental study of a methicillin-resistant Staphylococcus aureus epidemic in a burn unit. J Clin Microbiol 1983 Sep;18(3):683–688. PMID: 6630447. 10.1128/jcm.18.3.683-688.1983 PMC270875 6630447

[20] Allen KD, Green HD (1987). Hospital outbreak of multi-resistant Acinetobacter anitratus: an airborne mode of spread?. J Hosp Infect.

[21] Das I, Lambert P, Hill D (2002). Carbapenem-resistant Acinetobacter and role of curtains in an outbreak in intensive care units. J Hosp Infect.

[22] Roberts K, Smith CF, Snelling AM (2008). Aerial dissemination of Clostridium difficile spores. BMC Infect Dis.

[23] Prussin AJ, Schwake DO, Marr LC (2017). Ten questions concerning the aerosolization and transmission of Legionella in the built environment. Build Environ.

[24] Winn WC (1988). Legionnaires disease: historical perspective. Clin Microbiol Rev.

[25] Lee JY (2016). Tuberculosis infection control in health-care facilities: environmental control and personal protection. Tuber Respir Dis.

[26] Li Y, Haung X, Yu IT (2005). Role of air distribution in SARS transmission during the largest nosocomial outbreak in Hong Kong. Indoor Air.

[27] Improving the health of workers in indoor environments: priority research needs for a national occupational research agenda. Am J Public Health 2002; 92(9): 1430–1440. PMID: 12197969. 10.2105/ajph.92.9.1430. 10.2105/ajph.92.9.1430 PMC1447254 12197969

[28] Chen Y, Xu X, Liang J (2013). Relationship between climate conditions and nosocomial infection rates. Afr Health Sci.

[29] Morawska L (2006). Droplet fate in indoor environments, or can we prevent the spread of infection?. Indoor Air.

[30] Li Y, Leung GM, Tang JW (2007). Role of ventilation in airborne transmission of infectious agents in the built environment - a multidisciplinary systematic review. Indoor Air.

[31] Kowalski WJ. Air-treatment systems for controlling hospital-acquired infections. HPAC Engineering 2007;2(79):22. Accessed through: https://www.researchgate.net/publication/284450012.

[32] Beggs CB, Kerr KG, Noakes CJ (2008). The ventilation of multiple-bed hospital wards: review and analysis. Am J Infect Control.

[33] Costa A, Porretta A, Totaro M (2019). Microbiological air quality in heating, ventilation and air conditioning systems of surgical and intensive care areas: the application of a disinfection procedure for dehumidification devices. Pathogens.

[34] Seppänen OA, Fisk WJ (2004). Summary of human responses to ventilation. Indoor Air Suppl.

[35] Warris A, Verweij PE (2005). Clinical implications of environmental sources for Aspergillus. Med Mycol.

[36] Sehulster L, Chinn RY (2003). Guidelines for environmental infection control in health-care facilities. Recommendations of CDC and the Healthcare Infection Control Practices Advisory Committee (HICPAC). MMWR Recomm Rep..

[37] Medical Advisory Secretariat. Air cleaning technologies: an evidence-based analysis. Ontario Health Technology Assessment Series 2005; 5(17). PMID:23074468. PMC3382390 23074468

[38] Villafruela JM, Olmedo I, Berlanga FA (2019). Assessment of displacement ventilation systems in airborne infection risk in hospital rooms. PLoS One.

[39] Persily AK (2016). Field measurement of ventilation rates. Indoor Air.

[40] Pasquarella C, Pitzurra O, Savino A (2000). The index of microbial air contamination. J Hosp Infect.

[41] Dryden GE, Dryden SR, Brown DG (1980). Performance of bacteria filters. Respir Care.

[42] Maestre JP, Jennings W, Wylie D (2018). Filter forensics: microbiota recovery from residential HVAC filters. Microbiome.

[43] Memarzadeh F, Weiran X (2012). Role of air changes per hour (ACH) in possible transmission of air borne pathogens. Build Simul.

[44] Brock L (1975). The importance of environmetal conditions, especially temperature, in the operating room and intensive care ward. Br J Surg.

[45] Kalil AC, Metersky ML, Klompas M (2016). Management of adults with hospital-acquired and ventilator-associated pneumonia: 2016 clinical practice guidelines by the Infectious Diseases Society of America and the American Thoracic Society. Clin Infect Dis.

[46] Passaro L, Harbarth S, Landelle C (2016). Prevention of hospital-acquired pneumonia in non-ventilated adult patients: a narrative review. Antimicrob Resist Infect Control.

[47] Interim infection prevention and control recommendations for patients with suspected or confirmed coronavirus disease 2019 (COVID-19) in healthcare settings. Reference to website:https://www.cdc.gov/coronavirus/2019-ncov/infection-control/control-recommendations.html . Accessed 20 Mar 2020.

[48] World Health Organization: Infection prevention and control during health care when novel coronavirus (nCoV) infection is suspected: interim guidance. Reference to web site: https://www.who.int/docs/default-source/coronaviruse/clinical-management-of-novel-cov.pdf?sfvrsn=bc7da517_10&download=true. Accessed 20 Mar 2020.

[49] World Health Organization: Clinical management of severe acute respiratory infection (SARI) when COVID-19 disease is suspected: interim guidance. Reference to web site: https://apps.who.int/iris/handle/10665/331446. Accessed 20 Mar 2020.

[50] Alhazzani W, Moller MH, Arabi YM, et al. Surviving sepsis campaign: guidelines on the management of critically ill adults with coronavirus disease 2019 (COVID-19). Intensive Care Med 2020 Ahead of print. 10.1007/s00134-020-06022-5. 10.1007/s00134-020-06022-5 PMC7101866 32222812

[51] European Centre for Disease Prevention and Control. Novel coronavirus disease 2019 (COVID-19) pandemic: increased transmission in the EU/EEA and the UK – sixth update. Referenec to web site: https://www.ecdc.europa.eu/sites/default/files/documents/RRA-sixth-update-Outbreak-of-novel-coronavirus-disease-2019-COVID-19.pdf. Accessed 20 Mar 2020.

[52] Chan KH, Peiris JSM, Lam SY, et al. The effects of temperature and relative humidity on the viability of the SARS coronavirus. Adv Virol 2011. Article ID: 734690. PMID: 22312351. 10.1155/2011/734690. 10.1155/2011/734690 PMC3265313 22312351

[53] Casonova LM, Jeon S, Rutala WA (2010). Effects of air temperature and relative humidity on coronavirus survival on surfaces. Appl Environ Microbiol.

[54] Reijula J, Holopainen R, Kähkönen E (2013). Intelligent HVAC systems in hospitals. Intell Build Int.

[55] Abdul FSM, Mofarhi S, Abdulwahab Y (2015). The study of sustainable green HVAC systems in health care facilities. J Archit Eng Technol.

[56] Melikov AK (2004). Personalized ventilation. Indoor Air.

